# Are There Sex-Related Differences in the Effectiveness of Janus Kinase Inhibitors in Rheumatoid Arthritis Patients?

**DOI:** 10.3390/jcm13082355

**Published:** 2024-04-18

**Authors:** Cristina Martinez-Molina, Anna Feliu, Hye S. Park, Ana Juanes, Cesar Diaz-Torne, Silvia Vidal, Hèctor Corominas

**Affiliations:** 1 Department of Pharmacy, Hospital de la Santa Creu i Sant Pau, Sant Antoni Maria Claret 167, 08025 Barcelona, Spain; 2 Department of Medicine, Universitat Autònoma de Barcelona (UAB), Av. Can Domènech 737, 08193 Bellaterra, Spain; 3 Department of Rheumatology and Systemic Autoimmune Diseases, Hospital de la Santa Creu i Sant Pau, Sant Antoni Maria Claret 167, 08025 Barcelona, Spain; 4 Group of Immunology-Inflammatory Diseases, Institut de Recerca Sant Pau (IR SANT PAU), Sant Quintí 77-79, 08041 Barcelona, Spain

**Keywords:** rheumatoid arthritis, Janus kinase inhibitor, tofacitinib, baricitinib, upadacitinib, filgotinib, treat-to-target, treatment effectiveness, sex-related differences

## Abstract

**Background**: There is evidence suggesting the existence of sex differences in the effectiveness of specific drug classes for rheumatoid arthritis (RA). Our study stands as the first to elucidate sex-related differences in the effectiveness of Janus kinase (JAK) inhibitors. **Methods**: The study involved 150 RA patients treated with tofacitinib, baricitinib, upadacitinib, or filgotinib between September 2017 and October 2023. Sex differences in achieving remission and low disease activity (LDA) were identified through logistic regression analyses. Sex disparities in treatment effectiveness survival were evaluated through the Kaplan–Meier estimate, employing the log-rank test for comparison. The Cox model was applied to analyze the variable sex as a potential factor that could influence the maintenance of the JAK inhibitor treatment effectiveness. **Results**: Concerning the achievement of remission and LDA, no differences were observed between sexes in terms of the 28-joint Disease Activity Score (DAS28) C-reactive protein (CRP), the Clinical Disease Activity Index (CDAI), and the Simplified Disease Activity Index (SDAI). With respect to the DAS28-erythrocyte sedimentation rate (ESR), female patients, compared to males, possessed 70% lower odds of achieving remission (*p* = 0.018) and 66% lower odds of achieving LDA (*p* = 0.023). No differences were observed in treatment effectiveness survival between sexes (*p* = 0.703). Sex was not found to influence the survival of JAK inhibitor treatment effectiveness (*p* = 0.704). **Conclusions**: Being a female or male patient does not entail differences in the effectiveness of the JAK inhibitor treatment. Our findings encourage the consideration of a global pool of composite indices (DAS28-ESR/CRP, CDAI, SDAI) to measure RA disease activity, thus individualizing the target value as advocated by the treat-to-target strategy.

## 1. Introduction

Rheumatoid arthritis is a chronic inflammatory autoimmune disease that predominantly affects female more than male patients, typically represented in a 3:1 sex ratio [1]. Although the reason for this sexual disparity is not fully understood, multiple elements could potentially play a pathogenic role in rheumatoid arthritis.

According to the treat-to-target (T2T) strategy, the assessment of disease activity is required in routine clinical practice to guide treatment decisions [2,3]. To date, four validated composite measures are all applicable for this purpose. In 1995, the 28-joint Disease Activity Score (DAS28) was validated as a composite measure, using the erythrocyte sedimentation rate (ESR) as an inflammatory marker [4]. In 2004, the C-reactive protein (CRP) was included in the DAS28-CRP [5]. Finally, the Clinical Disease Activity Index (CDAI) and the Simplified Disease Activity Index (SDAI) emerged as the two other simplified validated measures, widely accepted in clinical practice [6]. Achieving clinical remission, or at least low disease activity, constitutes the primary target for rheumatoid arthritis treatment, as defined by the T2T recommendations [2,3] and advocated by several rheumatoid arthritis guidelines [7,8,9].

In light of these shared treatment targets between sexes, it has been suggested that female patients with rheumatoid arthritis tend to exhibit worse responses to biologic Disease-Modifying Antirheumatic Drugs (bDMARDs) compared to males [10,11,12]. Previous studies have considered being male as a predictive factor for achieving remission in the bDMARD treatment [13,14]. However, the magnitude of these sex disparities could differ depending on the specific drug class. Biological mechanisms related to sex, including immune profiles, could contribute to diverse treatment responses across the different rheumatoid arthritis drug classes.

**Figure 1:**
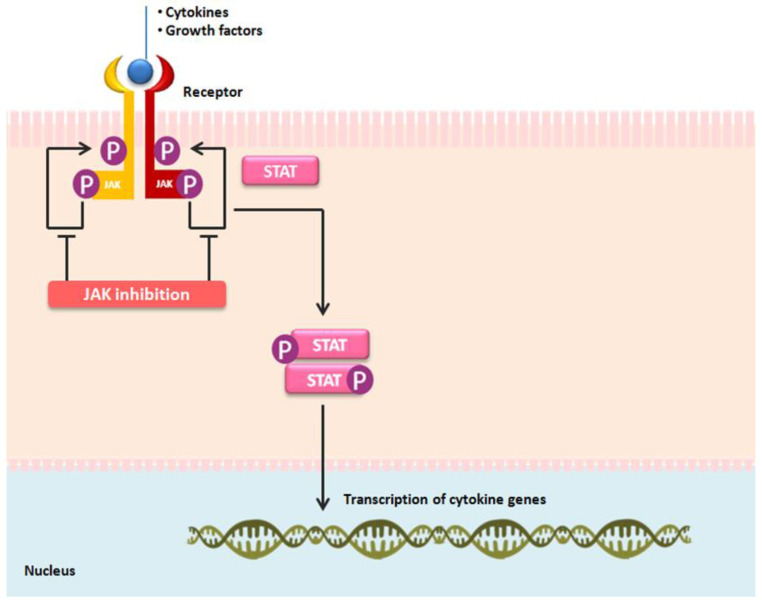
The mechanism of action of Janus kinase (JAK) inhibitors: tofacitinib, baricitinib, upadacitinib, and filgotinib. An extracellular recognition of cytokines and growth factors by their receptors leads to the intracellular phosphorylation of JAK enzymes. The involvement of specific JAKs (JAK1, JAK2, JAK3, and tyrosine kinase 2, TYK2) depends on their selective interactions with cytokine-receptor families. Activated JAKs phosphorylate the receptors, facilitating the recruitment and activation of signal transducer and activator of transcription (STAT) factors. Intracellular signals are transmitted through JAKs and seven STAT family members (STAT1-4, STAT5A, STAT5B, and STAT6) that promote transcription. JAK inhibitors mitigate cytokine effects by inhibiting the JAK-STAT signaling pathway.

The therapeutic arsenal for moderate to severe active rheumatoid arthritis has recently evolved to the regular use of targeted synthetic (ts) DMARDs (tsDMARDs), i.e., the Janus kinase (JAK) inhibitors, such as tofacitinib, baricitinib, upadacitinib, or filgotinib. Due to the fact that JAK inhibitors are the most recent drug class for rheumatoid arthritis treatment (Figure 1), it is notable how little published literature exists addressing sex disparities. The goal of the present study was to assess sex-related differences in the effectiveness of JAK inhibitor treatment in rheumatoid arthritis patients. The results may enhance the understanding of how sex, as a patient-related factor, might influence the JAK inhibitor treatment in rheumatoid arthritis.

## 2. Materials and Methods

### 2.1. Study Design and Patient Population

An observational retrospective study was conducted in a university hospital, which included real-world patients (aged ≥ 18 years) diagnosed according to the 2010 American College of Rheumatology (ACR)—European League Against Rheumatism (EULAR) classification criteria for rheumatoid arthritis [15]. All patients included were individually informed about the study protocol and were given the option to decline the extraction of data. Clinical data were collected from electronic medical records in October 2023. All patients were treated with either tofacitinib, baricitinib, upadacitinib, or filgotinib between September 2017 and October 2023.

### 2.2. Assessments

The exposure of interest of the study was focused on the sex of patients, categorized as female and male. The primary outcome was to assess sex differences in achieving both remission and low disease activity at the first 6 months of the JAK inhibitor treatment. This specific time frame, up to the first 6 months of treatment, was deemed appropriate for evaluating the primary outcome. According to the ACR-EULAR recommendations [7,8,9], if the T2T goal is not achieved within the first 6 months, a change in the treatment strategy should be considered. The secondary outcome was to determine sex disparities in the survival of the JAK inhibitor treatment effectiveness by analyzing the treatment retention. The retention of treatment was described as the time period from the treatment initiation and the definitive treatment discontinuation. With respect to disease activity, it was categorized based on the updated recommendations from the ACR [16], into remission, low disease activity, moderate disease activity, and high disease activity. Remission was determined by DAS28-ESR < 2.6, DAS28-CRP < 2.4, CDAI ≤ 2.8, and SDAI ≤ 3.3. Low disease activity was defined as DAS28-ESR < 3.2, DAS28-CRP < 2.9, and CDAI ≤ 10, SDAI ≤ 11.

### 2.3. Statistical Analyses

Demographic and clinical patient characteristics were separately detailed by sex. Differences between female and male patients were evaluated using the Mann–Whitney test (for ordinal or quantitative variables) and Fisher’s exact test (for categorical variables). Ordinal and quantitative variables are presented using the median and the interquartile range (IQR). Categorical variables are described as absolute number (n) and percentage (%).

The percentages of female and male patients achieving remission and low disease activity were determined at the first 6 months of the JAK inhibitor treatment. In order to evaluate the probability of attaining the study outcomes in female patients compared to males (control group), logistic regression analyses were conducted. Both crude and adjusted analyses for JAK inhibitor type, concomitant GC use, and concomitant csDMARD use were performed. All covariates that were statistically significant (*p* < 0.05) or exhibited borderline significance (*p* < 0.1 and >0.05) in the crude analyses were included in the adjusted analyses.

The JAK inhibitor retention due to the lack of treatment effectiveness was examined through the Kaplan–Meier estimate and the Cox proportional hazard regression model. The Kaplan–Meier estimate, for the discontinuation reason of lack of treatment effectiveness, was employed to evaluate the survival curves of female and male patients, with the log-rank test used for comparison. The bivariate Cox model was applied to analyze the variable sex as a potential factor that could influence that retention of the JAK inhibitor treatment.

The statistical analyses were performed utilizing STATA software version 12. A *p*-value of <0.05 was considered statistically significant.

### 2.4. Ethics Approval and Consent to Participate

Approval was obtained from the ethics committee of a hospital (IIBSP-JAG-2023-168). This study involving human participants was in accordance with the 1964 Helsinki declaration and its later amendments or comparable ethical standards.

## 3. Results

A total of 150 rheumatoid arthritis patients who received JAK inhibitor treatment were identified between September 2017 and October 2023. Their demographic and clinical characteristics are summarized in Table 1. No differences were observed in the JAK inhibitor type distribution between sexes. At JAK inhibitor treatment initiation, female and male patients presented comparable years of age, body mass index (BMI), years of disease duration, rheumatoid arthritis seropositivity considering rheumatoid factor (RF) and anti-cyclic citrullinated peptide (anti-CCP), prior conventional synthetic (cs) DMARD (csDMARD) use, and prior bDMARD use. Both sexes showed similar use of concomitant glucocorticoids (GC) and concomitant csDMARDs at the JAK inhibitor treatment initiation. In terms of disease activity, similar scores were noted between female and male patients, with the exception of the DAS28-ESR, which was higher in female patients, both at baseline (*p* = 0.043) and at the first 6 months of treatment (*p* = 0.014).

The main findings from the logistic regression analyses are shown in Table 2. Compared to males, female patients were less likely to achieve the DAS28-ESR remission [unadjusted odds ratio (OR): 0.32; 95% confidence interval (CI): 0.12–0.83; *p* = 0.019] or the DAS28-ESR low disease activity (OR: 0.34; 95% CI: 0.13–0.85; *p* = 0.022), at the first 6 months of the JAK inhibitor treatment. The multivariate model showed similar results, with adjusted odds ratio (ORadj) of 0.30 (95% CI: 0.11–0.81; *p* = 0.018) and 0.34 (95% CI: 0.13–0.86; *p* = 0.023), respectively. There were no significant differences between sexes in the achievement of remission and low disease activity for the DAS28-CRP, the CDAI, and the SDAI.

**Figure 2:**
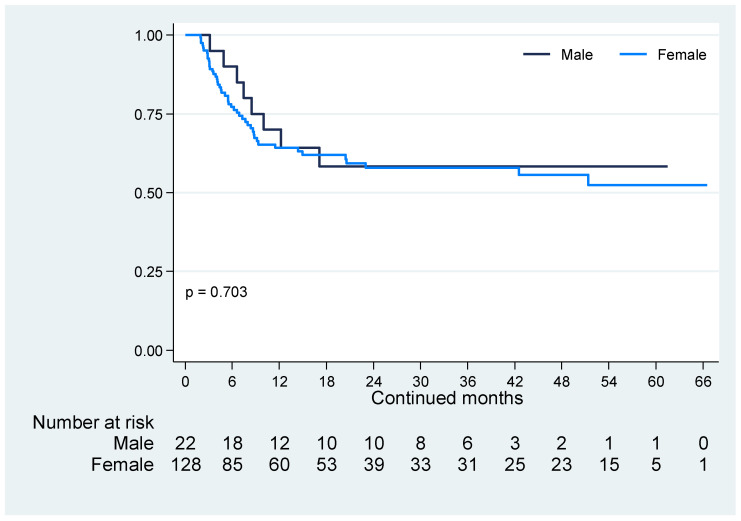
JAK inhibitor retention due to the lack of treatment effectiveness. Similar JAK inhibitor retentions due to the lack of treatment effectiveness were observed between female and male patients (*p* = 0.703) during the follow-up period from September 2017 to October 2023.

JAK inhibitor retention, for the discontinuation reason of lack of treatment effectiveness, is depicted in Figure 2. No differences were observed in retention rates between female and male patients (*p* = 0.703). Sex was not found to influence the survival of the JAK inhibitor treatment effectiveness [hazard ratio (HR) for female patients: 1.16; 95% CI: 0.55–2.45; *p* = 0.704].

## 4. Discussion

This study assessed sex-related differences in the effectiveness of tofacitinib, baricitinib, upadacitinib, and filgotinib in the clinical context of rheumatoid arthritis treatment. Taking into account the available literature, there are currently limited published real-world studies addressing this concern. Indirectly, previous studies have suggested that being either a female or male patient does not influence the survival of the JAK inhibitor treatment effectiveness [17,18,19]. To the best of our knowledge, this study stands as the first to elucidate sex-related disparities in the achievement of JAK inhibitor treatment effectiveness, while also shedding light on the survival of the effectiveness of these small molecules.

In terms of achieving remission and low disease activity at the first 6 months of the JAK inhibitor treatment, no significant differences between sexes were observed concerning the DAS28-CRP, the CDAI, or the SDAI. With respect to the DAS28-ESR, female patients, compared to males, possessed 70% lower odds of achieving remission (ORadj for female: 0.30; 95% CI: 0.11–0.81; *p* = 0.018) and, similarly, 66% lower odds of achieving low disease activity (ORadj for female: 0.34; 95% CI: 0.13–0.86; *p* = 0.023). ESR and CRP are acute-phase reactants commonly used in routine clinical practice as inflammation biomarkers, with ESR levels typically being higher in female than in male patients [20,21,22]. In this manner, sex disparities in ESR levels were reflected in the DAS28-ESR values both at baseline (female: 5.4 [4.8–6.1]; male: 5 [4–5.5]; *p* = 0.043) and at 6 months of the JAK inhibitor treatment (female: 3.7 [2.9–5.2]; male: 3 [1.7–4.4]; *p* = 0.014), leading to the misclassification in female patients of both the DAS28-ESR remission and the DAS28-ESR low disease activity. In accordance with the foregoing, our study findings suggest that, specifically for JAK inhibitor treatment, being a female or male patient does not influence the achievement of remission or low disease activity.

In terms of the survival of the JAK inhibitor treatment effectiveness, Figure 2 illustrates the treatment strategy selected based on the clinical judgment of our clinical rheumatologists in response to the lack of treatment effectiveness. According to both the T2T strategy [2,3] and the rheumatoid arthritis guidelines [7,8,9], if substantial improvement in disease activity is not plausible within the first 3 months of treatment, or if the primary target remains unattained by 6 months, treatment adjustment or modification of the therapy is recommended. The unobserved significant differences between female and male patients in the JAK inhibitor treatment retention rates (*p* = 0.703), as well as the non-determination of sex as a potential factor that could influence the maintenance of the treatment effectiveness (HR: 1.16; 95% CI: 0.55–2.45; *p* = 0.704), suggest that being a female or male patient does not influence the maintenance of the effectiveness of these small molecules.

The present study had some inherent limitations. The first of these was the population size and that it was exclusively conducted at a single healthcare center. However, the results obtained align with previous indirect data regarding discrepancies in the survival of the JAK inhibitor treatment effectiveness between female and male patients. Second was the disparity in sex distribution, reflecting real-world clinical data of a disease that predominantly affects females over males. Third, due to the retrospective nature of this study, despite adjusting for potential confounders, there remains the possibility that treatment retention may have been influenced by unmeasured cofounders not accounted for in our adjusted models. Fourth, JAK inhibitors other than tofacitinib, baricitinib, upadacitinib, or filgotinib —the four small molecules currently approved in Europe for the treatment of rheumatoid arthritis—were not considered in our study, i.e., peficitinib. Fifth, adjustments in dosage or frequency of drug treatment were not monitored in our study. JAK inhibitors are approved at specific dosages, with certain treatment adjustments recommended based on specific patient characteristics and concomitant treatments. We assumed that all patients received their required dosage and frequency in accordance with the approved recommendations for JAK inhibitor treatment. Sixth, it would be interesting to precisely understand the influence of sex on the components of the four validated disease activity measures; however, data regarding this aspect were lacking in the present study.

The main strength of our study lies in the inclusion of rheumatoid arthritis patients undergoing treatment in real-world clinical settings, with the primary aim of elucidating whether sex-related disparities exist that could influence the achievement and survival of the JAK inhibitor treatment effectiveness, thereby establishing the initial findings on this matter within the published literature.

## 5. Conclusions

To sum up, the findings of our study suggest that being a female or male patient does not entail differences in the effectiveness of the JAK inhibitor treatment, taking into account the potential misclassification in female patients for both the DAS28-ESR remission and the DAS28-ESR low disease activity, attributable to sex disparities in the ESR levels. This study encourages the consideration of a global pool of validated composite indices (DAS28-ESR, DAS28-CRP, CDAI, and SDAI) to measure rheumatoid arthritis disease activity, thus individualizing the target value based on patient-related factors, as advocated by the T2T strategy [2,3].

## Figures and Tables

**Table 1:** Demographic and clinical patient characteristics.

Parameters	Female (n = 128)	Male (n = 22)	*p*-Value
*Age* (years), median [IQR]	65 [52–71]	61 [53–75]	0.855
*BMI* [weight(kg)/height(m^2^)], median [IQR]	27.0 [24.0–30.3]	27.9 [25.4–30.7]	0.407
*RA disease duration* (years), median [IQR]	14.0 [5.0–24.5]	9.5 [5.0–19.0]	0.126
*RA seropositivity*, n (%)			
RF	77 (60.2)	15 (68.2)	0.636
Anti-CCP	96 (75.0)	17 (77.3)	1.000
*Previous csDMARDs*, n (%)			
Methotrexate	127 (99.2)	22 (100)	1.000
Leflunomide	62 (48.5)	8 (36.4)	0.358
Sulfasalazine	32 (25.0)	5 (22.7)	1.000
Other csDMARDs	50 (39.1)	5 (22.7)	0.159
*Previous bDMARDs*, n (%)			
Adalimumab	63 (49.2)	10 (45.5)	0.820
Certolizumab	41 (32.0)	7 (31.8)	1.000
Etanercept	51 (39.9)	10 (45.5)	0.645
Golimumab	27 (21.1)	4 (18.2)	1.000
Infliximab	22 (17.2)	1 (4.6)	0.200
Tocilizumab	65 (50.8)	7 (31.8)	0.112
Sarilumab	26 (20.3)	2 (9.1)	0.372
Abatacept	56 (43.8)	7 (31.8)	0.355
Rituximab	32 (25.0)	2 (9.1)	0.165
*JAK inhibitor type*, n (%)			0.198
Tofacitinib	40 (31.3)	11 (50.0)	
Baricitinib	69 (53.9)	7 (31.8)	
Upadacitinib	9 (7.0)	2 (9.1)	
Filgotinib	10 (7.8)	2 (9.1)	
*Concomitant GC use*, n (%)	79 (61.7)	14 (63.6)	1.000
PDN dose (mg/day), median [IQR]	5.0 [0.0–5.0]	5.0 [0.0–5.0]	0.671
*Concomitant csDMARD use*, n (%)	35 (27.3)	8 (36.4)	0.446
Methotrexate	24 (18.8)	5 (22.7)	0.770
Leflunomide	1 (0.8)	2 (9.1)	0.056
Sulfasalazine	5 (3.9)	0 (0.0)	1.000
Other csDMARD	5 (3.9)	1 (4.6)	1.000
*RA disease activity at baseline*, median [IQR]			
DAS28-ESR	5.4 [4.8–6.1]	5.0 [4.0–5.5]	0.043
DAS28-CRP	4.7 [4.1–5.3]	4.6 [4.1–5.1]	0.572
CDAI	25.5 [19.5–32.0]	19.0 [16.0–26.0]	0.098
SDAI	25.6 [18.0–31.5]	20.3 [16.2–26.2]	0.288
*RA disease activity at 6 months*, median [IQR]			
DAS28-ESR	3.7 [2.9–5.2]	3 [1.7–4.4]	0.014
DAS28-CRP	2.9 [1.9–4.3]	2.3 [1.6–4.2]	0.232
CDAI	10 [5.0–23.5]	6.5 [4.0–17.0]	0.093
SDAI	10.2 [5.2–23.2]	5.7 [3.1–18.6]	0.091

IQR—interquartile range [P25-P75], BMI—body mass index, RA—rheumatoid arthritis, RF—rheumatoid factor, anti-CCP—anti-cyclic citrullinated peptide, csDMARD—conventional synthetic Disease Modifying Anti-Rheumatic Drug, bDMARD—biologic Disease Modifying Anti-Rheumatic Drug, JAK—Janus kinase, GC—glucocorticoid, PDN—prednisone, DAS28-ESR—Disease Activity Score 28-joint count using Erythrocyte Sedimentation Rate, DAS28-CRP—Disease Activity Score 28-joint count using C-Reactive Protein, CDAI—Clinical Disease Activity Index, SDAI—Simplified Disease Activity Index.

**Table 2:** Logistic remission analyses examining the effect of sex on the study outcomes.

Outcomes at 6 Months	Female, n (%)	Male, n (%)	OR for Female [95% CI]	*p*-Value	ORadj for Female [95% CI]	*p*-Value
DAS28-ESR remission	23 (18.0)	9 (40.9)	0.32 [0.12–0.83]	0.019	0.30 [0.11–0.81]	0.018
DAS28-ESR LDA	42 (32.8)	13 (59.1)	0.34 [0.13–0.85]	0.022	0.34 [0.13–0.86]	0.023
DAS28-CRP remission	44 (34.4)	11 (50.0)	0.52 [0.21–1.30]	0.165	0.52 [0.20–1.32]	0.169
DAS28-CRP LDA	61 (47.7)	13 (59.1)	0.63 [0.25–1.58]	0.324	0.61 [0.24–1.58]	0.309
CDAI remission	9 (7.0)	3 (13.6)	0.48 [0.12–1.93]	0.301	0.47 [0.11–1.98]	0.305
CDAI LDA	67 (52.3)	14 (63.6)	0.63 [0.25–1.60]	0.329	0.58 [0.22–1.58]	0.289
SDAI remission	20 (15.6)	6 (27.3)	0.49 [0.17–1.41]	0.189	0.54 [0.18–1.62]	0.273
SDAI LDA	68 (53.1)	13 (59.1)	0.78 [0.31–1.97]	0.605	0.74 [0.28–1.95]	0.546

OR—odds ratio, CI—confidence interval, ORadj—odds ratio adjusted, DAS28-ESR—Disease Activity Score 28-joint count using Erythrocyte Sedimentation Rate, LDA—low disease activity, DAS28-CRP—Disease Activity Score 28-joint count using C-Reactive Protein, CDAI—Clinical Disease Activity Index, SDAI—Simplified Disease Activity Index.

## Data Availability

The datasets used and/or analyzed during the current study are available from the corresponding author on reasonable request.
